# Molecular Epidemiology and Co-Circulation of Foot-and-Mouth Disease Serotypes in Ghana, 2024

**DOI:** 10.3390/v18070769

**Published:** 2026-07-13

**Authors:** Theophilus Odoom, Sherry Ama Mawuko Johnson, William Tasiame, Richard Kwamena Abbiw, Kingsley Kwabena Amoako, Benita Anderson, Joseph K. Abuh, Kofi Sarpong, Fenteng Danso, Emmanuel Allegye-Cudjoe, Lizhe Xu, Amy Berninger, Corrie Brown, Bonto Faburay

**Affiliations:** 1 Accra Veterinary Laboratory, Veterinary Service Directorate, Accra P.O. Box M161, Ghana; theodoom@gmail.com (T.O.); kamoako15@gmail.com (K.K.A.); skofi5961@gmail.com (K.S.); 2 School of Veterinary Medicine, University of Ghana, Accra P.O. Box LG 25, Ghana; 3 School of Veterinary Medicine, Kwame Nkrumah University of Science and Technology, Kumasi, Ghana; drwilly2002@gmail.com; 4 West African Centre for Cell Biology of Infectious Pathogens, University of Ghana, Accra P.O. Box LG 25, Ghana; abbiw.richardk@gmail.com; 5 Tsetse and Trypanosomiasis Control, Veterinary Service Directorate, Accra P.O. Box 97, Ghana; btdjagmah@yahoo.com; 6 Veterinary Service Directorate, Accra P.O. Box M161, Ghana; josephabuh@yahoo.com (J.K.A.); yfenteng@yahoo.com (F.D.); emmallec@gmail.com (E.A.-C.); 7 Foreign Animal Disease Diagnostic Laboratory (FADDL), National Veterinary Services Laboratories, Animal and Plant Health Inspection Service, United States Department of Agriculture, National Bio and Agro-Defense Facility, Manhattan, KS 66505, USA; lizhe.xu@usda.gov (L.X.); amy.berninger@usda.gov (A.B.); 8 LifeStock International, Athens, GA 30606, USA; corrie@lifestock.org

**Keywords:** foot-mouth-disease, Ghana, point of entry, probang, FMD virus serotypes

## Abstract

Foot-and-mouth disease (FMD) represents one of the most severe transboundary animal diseases globally. Although considered endemic in Ghana, recent data on circulating serotypes are limited. This study investigated and characterized FMD outbreaks occurring between July and December 2024, assessed the occurrence of the infection in seemingly healthy cattle imports, and explored alternative methods to enhance early diagnosis in livestock; using probang Sera, epithelial tissue and oral mucosal swabs were obtained from cattle exhibiting signs suggestive of FMD from farms, livestock markets and a slaughter facility. This was followed by a cross-sectional survey at two major points of entry (POE) to Ghana to obtain oropharyngeal samples using probang from healthy cattle to assess the possible role of imported cattle in the outbreaks in the northern and southern regions of the country. Samples were analysed for FMD virus (FMDV) and non-structural protein (NSP) antibodies to assess infection and exposure from the outbreak foci and POE. Positive samples were sequenced to characterize the virus serotypes. Outbreaks were reported in 38 kraals, three livestock markets, and one slaughter facility across 14 districts in nine regions. Morbidity rates ranged from 8% to 100% (median = 50%), with fatalities occurring in three kraals. Of the 247 samples from outbreak locations, FMD virus was detected in 49.4% (122/247). A majority of cattle (66.03%, 140/212) tested seropositive for FMD NSP antibodies. From healthy cattle at POE, 250 oropharyngeal (probang) samples were collected, with 62.4% testing positive, distributed between the north and south. Only 1.6% of the samples had FMDV RNA detected using PCR. Phylogenetic analysis identified serotypes O, A, and SAT2. These findings confirm the co-circulation of multiple FMDV serotypes in Ghana and highlight the necessity for enhanced molecular surveillance and coordinated cross-border control measures.

## 1. Introduction

Foot-and-mouth disease (FMD) is a transboundary animal disease (TAD) that predominantly affects cloven-hoofed animals [1]. The causative agent is the foot-and-mouth disease virus (FMDV), a single-stranded positive-sense RNA virus approximately 8.4 kb long [1,2]. The FMDV belongs to the genus *Aphthovirus* of the *Picornaviridae* family and comprises seven immunologically distinct serotypes that do not provide cross immunity. The serotypes are O, A, C, Asia 1, and Southern African Territories (SAT) -1, SAT-2, and SAT-3 [3]. Foot-and-mouth disease (FMD), although effectively controlled in most regions of Europe and America, remains prevalent in areas with insufficient surveillance systems, particularly in developing countries in Africa, the Middle East, and Asia. [4]. The disease is highly contagious and may persist in the environment for a period of time in the presence of organic material, complicating control efforts [5]. Although the mortality rates are low, FMD significantly affects livestock productivity and rural livelihoods, particularly in low- and middle-income countries [4,6].

The disease is responsible for annual economic losses estimated at $6.5 billion [6] and has been targeted for eradication following the successful elimination of rinderpest [7]. In Africa, where six of the seven known serotypes are prevalent, the World Bank ranks FMD as the third most impactful livestock disease contributing to poverty in the region [4]. Smallholder farmers, who form the backbone of rural economies, are disproportionately affected by a reduced milk yield, decreased weight gain, poor feed conversion efficiency, and limited access to trade [8]. Although these impacts are often visible, they are difficult to quantify and contribute to insecurities in long-term livelihoods. A significant challenge in controlling FMD is the carrier state, wherein approximately 50–60% of infected livestock may retain the virus in the oropharynx for more than 28 days post-infection [9]. These animals can act as reservoirs, potentially transmitting the virus to susceptible populations, including young animals, where the outcomes are frequently fatal.

The disease is endemic to West Africa, with every country reporting at least one outbreak annually [3]. In these endemic nations, including Ghana, FMD is often underreported and misdiagnosed due to the clinical resemblance to other diseases [10]. Moreover, a lack of molecular epidemiological studies and serotype characterization has led to gaps in the understanding of the viral dynamics and control strategies [3,10]. Inadequate surveillance systems and animal movement within and between countries through trade and communal grazing during drought seasons contribute to persistent FMD outbreaks [3,4,11]. Understanding the molecular epidemiology and circulating serotypes of FMDV in Ghana is crucial for informing control strategies and policies, contributing to regional eradication efforts, and reducing the economic burden of the disease.

This study aimed to investigate and characterize the serotypes of the FMDV in outbreak settings, assess the prevalence of FMD in seemingly healthy cattle imports, and explore alternative methods to enhance the early diagnosis of FMD in livestock using probangs.

## 2. Materials and Methods

### 2.1. Sampling

#### 2.1.1. Outbreaks Investigation

All outbreaks reported between July (rainy season) and December (dry season) 2024 were investigated within 48 h of notification, and samples were collected to confirm the outbreak and characterize the virus. Sera, epithelial tissue, and oral mucosal swabs were obtained from farms, livestock markets, and holding pens at slaughter facilities. These samples were tested using serological assays and real-time PCR.

#### 2.1.2. Survey—Study Area and Samples

Another set of samples was obtained from two border entry points to Ghana in the northern and southeastern regions of the country. Ghana shares its northern border with Burkina Faso, where Paga serves as the primary entry point for livestock. In the east, Ghana borders Togo, with Dzodze serving as the formal entry point for the livestock. We collected samples from Tulaku, a livestock market in the Greater Accra region, which serves as a hub for livestock from all entry points.

Samples obtained from the animals exhibiting clinical signs suggestive of FMD included sera (250) and epithelial tissue (buccal and pedal) from 83 cattle. Oral mucosal swabs were analyzed using serological assays and real-time PCR. A total of 250 oropharyngeal samples were collected, using probang, from apparently healthy cattle arriving at the border and livestock market. A probang is a flexible steel rod with a cup or sponge at the end, which is used to collect epithelial cells and fluid from the oropharyngeal region near the tonsils. This method detects the FMD virus in carriers and persistently infected animals without visible clinical signs of infection. In addition, 20 environmental samples were collected. Swabs moistened with virus transport medium (VTM) were used to swab the truck floors transporting the animals.

### 2.2. Molecular Detection

#### 2.2.1. Epithelium Sample Preparation

Approximately 1.0 g of epithelial and vesicular tissues, initially preserved in Viral Transport Medium (Liofilchem, Roseto degli Abruzzi, Italy), were ground with 0.5 g of sterile sand using a mortar and pestle within a Class II biosafety cabinet in a BSL-3 modular laboratory. This mixture was subsequently resuspended in 9 mL of phosphate-buffered solution (PBS) to create a 10% tissue homogenate. The solution was centrifuged at 2000 rpm for 5 min at room temperature, and the supernatant was collected for RNA extraction.

#### 2.2.2. RNA Extraction

Viral RNA was extracted using the Qiagen RNeasy kit (QIAGEN, Hilden, Germany) according to the manufacturer’s instructions. In brief, approximately 10% (140 µL) of the tissue homogenate was used for extraction. The RNA was eluted in a final volume of 60 µL of elution buffer and stored at +4 °C for immediate use or at −80 °C until used for real-time reverse transcription–polymerase chain reaction (qRT-PCR).

#### 2.2.3. Real-Time Polymerase Chain Reaction (qRT-PCR) Amplification

Extracted RNA samples were tested for the presence of FMDV according to the protocol described by Callahan et al. [12]. This one-step real-time RT-PCR detects the 3D RNA polymerase encoding gene with primer sequences as follows: forward primer (FMD^3D^F) 5′ACT GGG TTT TAC AAA CCT GTG A; reverse primer (FMD^3D^R) 5′GCG AGT CCT GCC ACG GA; probe (TagMan probe); TCC TTT GCA CGC CGT GGG AC. The probe was labelled with a 5′-reporter dye, 6-carboxyfluorescein, and a 3′-quencher, tetramethylrhodamine, to detect all FMDV in a single-tube reaction.

#### 2.2.4. Sequencing

Fifty-six samples with ct values below 25 were submitted to the National Veterinary Services Laboratories’ Foreign Animal Disease Diagnostic Laboratory (FADDL), Manhattan USA, and nucleic acids were extracted using a MagMax CORE Nucleic Acid Purification kit (Thermo Fisher, Waltham, MA, USA) following the manufacturer’s protocol, except for the final elution step, which used 70 µL instead of 90 µL of elution buffer. Extracted RNAs were tested by FMDV RT-qPCR [12]. Five RT-qPCR positive samples were selected for Direct RNA Sequencing [13] on GridION (Oxford Nanopore Technologies, ONT, Oxford, UK) using an SQK-RNA004 kit and FLO-MIN004RA RNA flow cell. The 55 RT-qPCR positive RNA samples were converted to cDNA using the SISPA method [14], and the resulting cDNA were sequenced with Illumina DNA Prep kit on the MiSeq (Illumina, San Diego, CA, USA) and with the ONT Rapid PCR Barcoding Kit (RPB114.24) on the GridION to get the full genome sequences. Fourteen of the 55 samples were further analyzed by target-specific amplification using an FMDV universal primer [15], which spans the region encoding the leader protease through 2A. After purification with Ampure XP beads (Backman, Coulter Life Sciences, Indianapolis, Indiana, United State), the amplicons were sequenced using the Illumina DNA Prep kit on the MiSeq and the ONT Rapid Barcoding Kit (SQK-RBK114.96) on GridION.

The data generated from both Illumina and ONT were assembled with CLC Genomics Workbench (Qiagen, Hilden, Germany) version 25.0.3, using the De Novo Assemble Long Reads and Polish with Short Reads workflow and De Novo with Short Reads. After analyzing the contigs with the NCBI Blast tool online to identify FMDV-related contigs, the top-hit isolate from the Blast result for each positive sample was downloaded and used as a reference for the given sample to build the reference-based contig with both ONT and Illumina reads or Illumina reads only. The contigs from both platforms were compared, and the consensus sequence was created for the sample.

The samples with full length VP1 sequences were selected for phylogenetic analysis. The sequences of the FMDV topotype representative isolates of serotype A, O and Sat 2 were downloaded from Pirbright Institute (https://www.wrlfmd.org/fmdv-genome/fmd-prototype-strains#panel-8393, accessed 20 February 2026). The multiple sequence alignments of each of the three serotypes between the downloaded isolates and Ghana samples in this study were tested with the Model Testing tool in CLC to assess the best model to use in the Maximum Likelihood Phylogeny tool. Based on the result, a Maximum Likelihood phylogenetic tree was constructed in CLC with 1000 bootstraps.

### 2.3. Detection of FMDV NSP Antibodies

Sera were tested for antibodies against the non-structural protein (NSP) of FMDV using a commercial competitive ELISA kit (ID Screen^®^ FMD NSP, IDVet, 310, Rue Louis Pasteur, Montpellier, France). The assay was performed according to the manufacturer’s instruction. The optical density (OD) was measured at 450 nm using Multiskan skyhigh (2024), and the competition percentage (S/N%) was calculated as

$$\frac{S}{N}\%=\frac{{OD}_{sample}}{{OD}_{NC}}\times 100$$

The assay was considered valid if the mean negative control OD (OD_NC_) was >0.7, and the mean positive control OD (OD_PC_) was <30% of the OD_NC_. Samples with S/N% ≤ 50% were interpreted as positive, while those with S/N% > 50% were negative.

## 3. Results

### 3.1. Outbreak Investigation

**Figure 1:**
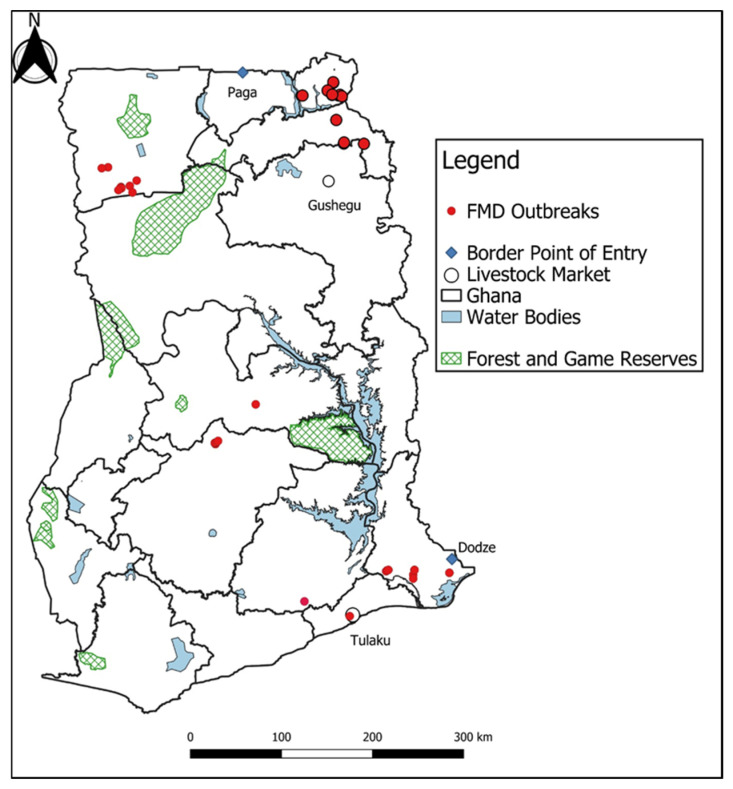
Map of Ghana showing the outbreak sampled sites.

Outbreaks of FMD were reported in 38 kraals, three livestock markets, and a holding area of a slaughterhouse across 14 districts within 9 of the 16 administrative regions in Ghana (Figure 1). The herd sizes in the kraals ranged from 10 to 300, with a median of 70. A total of 247 cattle and four sheep were tested for the presence of FMD virus (FMDV) and non-structural protein (NSP) antibodies. Morbidity was observed on all farms, with rates ranging from 8.0% to 100% and a median of 50%. Case fatality rates were documented in three kraals (7.9%; 3/38) in the Volta region. Two livestock markets sampled were Ashaiman Tulaku (in the south) and Gushegu (in the north). All four of the cattle sampled from Tulaku were FMDV-positive (100%). In Gushegu market, six of nine samples were positive (66.7%).

**Figure 2:**
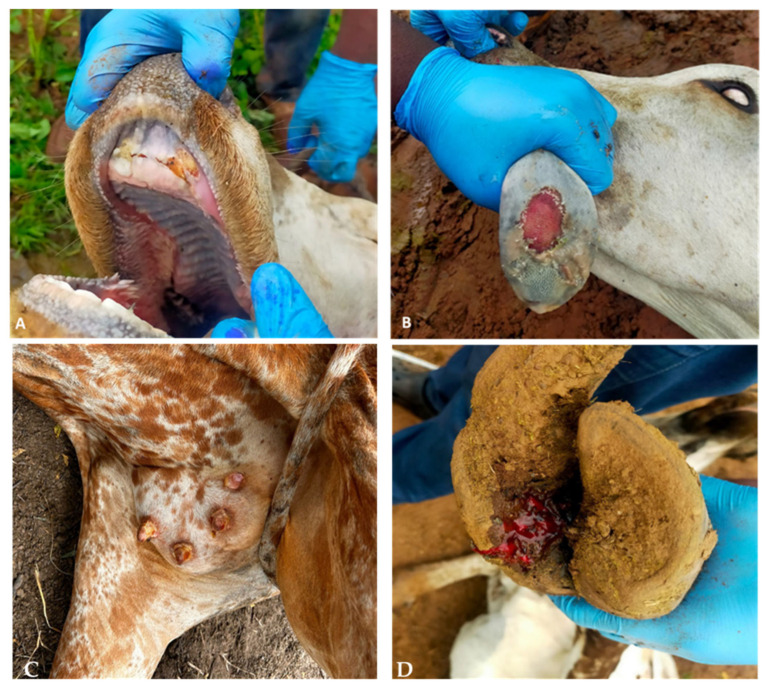
Foot-and-mouth disease (FMD) lesions observed on affected cattle on a farm in West Akim, Eastern Region, Ghana. Ulcers on the (**A**) gums and (**B**) tongue of an affected cow. (**C**) shows erosions and ulcerations on the teats of a cow. (**D**) shows ulcerations on the hoof of a bull.

The main clinical signs exhibited by the affected animals included drooling due to the presence of vesicles and ulcers in the buccal cavity (Figure 2A,B). Sores and vesicles were also observed on the udders of some cows (Figure 2C). Lameness was observed in some animals, attributed to the presence of vesicles and wounds located at the coronary band of the hooves, heels, and interdigital spaces (Figure 2D). Marked emaciation was observed, particularly in calves.

#### 3.1.1. Presence of FMDV NSP Antibodies

**Figure 3:**
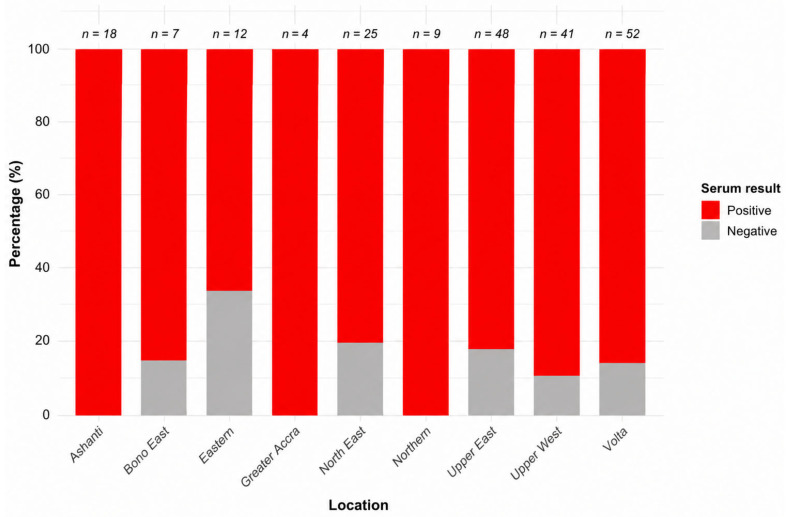
Distribution of sampled cattle sera across regions showing proportions positive and negative for foot-and-mouth disease (FMD) non-structural protein (NSP) antibodies.

Of the 212 serum samples collected, 66.03% (140) tested positive for FMDV NSP, as illustrated in Figure 3. The NSP antibodies were determined using a commercial competitive ELISA kit (ID Screen^®^ FMD NSP, Montpellier, IDVet, France) with a competition percentage ≤ 50% considered positive. All samples were obtained within 48 h post-reporting of outbreaks. Higher proportions of sera were positive for FMD NSP irrespective of the location.

#### 3.1.2. Presence of FMDV

**Figure 4:**
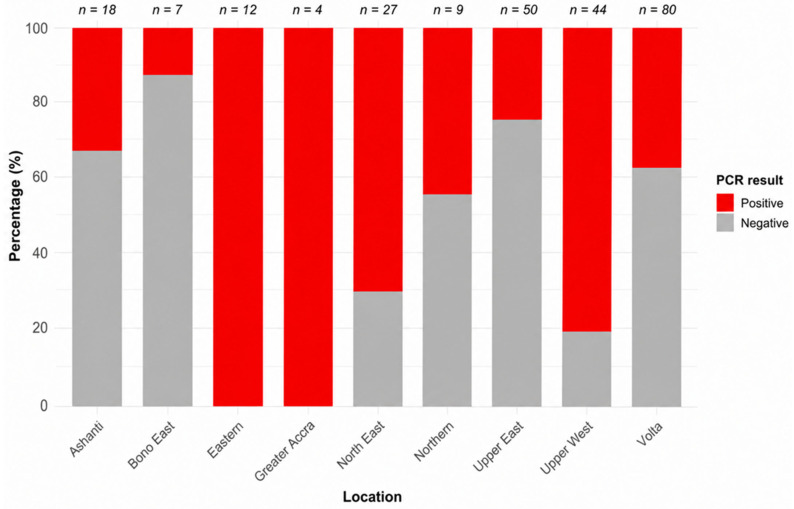
Distribution of foot-and-mouth disease virus (FMDV) in samples with respect to region. Epithelial samples obtained from cattle from suspected FMD outbreaks were tested for FMDV using qRT-PCR.

Among the 247 cattle sampled across the nine regions, FMDV RNA was detected in 122 (49.4%) by PCR (Figure 4). Four regions reported positive rates exceeding 60%. Two of these, Upper West and North East, are located in the northern part of the country, while the other two, Greater Accra and Eastern, are in the southern part of the country. Both southern regions recorded a 100% positive rate. The region with the lowest positive percentage was Bono East, with only 14.3%.

Animals were sampled within 48 ± 3.5 h after an outbreak had been reported. The low concordance could be due to the samples being collected during the first few days after infection, when antibodies to NSP may not have yet developed.

#### 3.1.3. FMDV Sequence and Phylogenetic Analysis

Among the 56 samples received, 55 samples were positive for FMDV RT-qPCR [12], with Ct values ranging from 11.80 to 36.99; one sample was negative. Five samples—each representing one of the five sampling regions (Volta, Upper West, North East, Upper East and Greater Accra)—and having the lowest Ct values (from 11.80 to 20.88) were sequenced using Direct RNA Sequencing [13] with ONT FLO-MIN004RA kit on the GridION. Next-generation sequencing data from both ONT and Illumina platforms were combined and analyzed. This resulted in the recovery of eleven full genomes, including one serotype A, three serotypeSAT 2, and seven serotype O viruses. These sequences have been submitted to GenBank (accession numbers PX864599 to PX864609, to be released upon publication). In addition, three extra samples yielded partial genome but complete VP1 sequences, all belonging to serotype O. Comparison of the VP1 sequence of the ten serotypes O samples showed that they represented four unique sequence types: two were unique to a single sample (Gha_20 and Gha_25; GenBank PZ225554-PZ225555), one was shared by three samples (Gha_15, Gha_19, and Gha_Ashfmd1, labeled Gha_x3 in the phylogenetic analysis), and one was shared by five samples (Gha_LM-15, LMF40-12, LMF40-17, LMF80 and LMM30, labeled Gha_LMx5).

**Figure 5:**
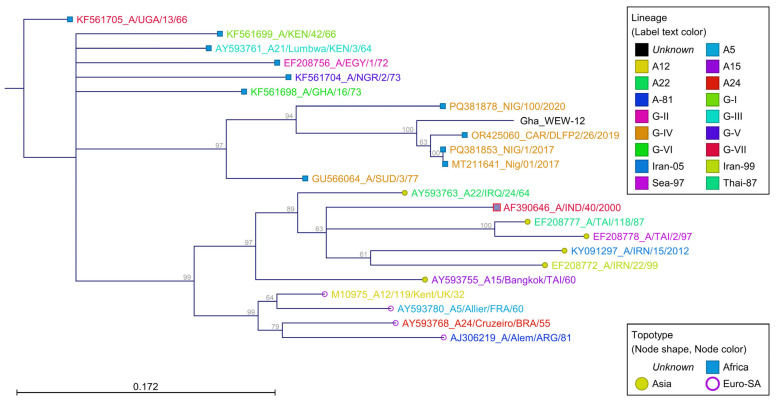
Phylogenetic analysis of foot-and-mouth disease virus (FMDV) serotype A isolates showing relationships among global lineages and topotypes. Node shape and color represent viral topotype, isolate labels color represents viral lineage. Ghanaian isolates are labelled with “Gha_”. The A isolates Gha_WEW-12 clusters within the Africa topotype and is closer to G-IV lineage. The phylogenetic tree was constructed using maximum likelihood methods based on VP1 sequences with the General Time Reversible substitution model with gamma distributed rates across sites (four categories). The scale bar represents the number of nucleotide substitutions per site. Bootstrap values are shown on the trees with a threshold of 60%.

**Figure 6:**
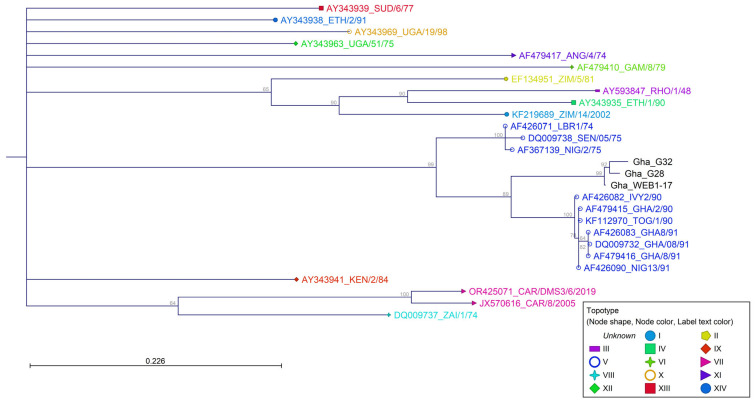
Phylogenetic analysis of foot-and-mouth disease virus (FMDV) serotype SAT 2 isolates showing relationships among global lineages and topotypes. Node shape and color, as well as label text color represent viral topotype; Ghanaian isolates are labelled with “Gha_”, and all three isolates (G32, G28 and WEB1-17) are clustered together, all belonging to topotype V. The tree was constructed using maximum-likelihood methods based on VP1 sequences with the General Time Reversible substitution model with gamma-distributed rates across sites (four categories). The scale bar represents the number of nucleotide substitutions per site. Bootstrap values are shown on the trees with a threshold of 60%.

**Figure 7:**
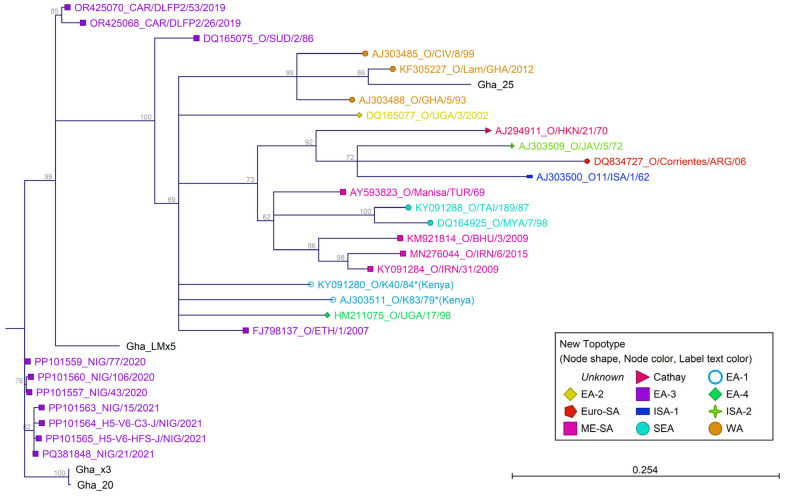
Phylogenetic clustering of foot-and-mouth disease virus (FMDV) serotype O isolates. Topotype groupings are indicated by node shape and color as well as label color. The tree was inferred using maximum-likelihood analysis of VP1 nucleotide sequences with the General Time Reversible substitution model with gamma-distributed rates across sites (four categories). The scale bar denotes nucleotide substitutions per site. Bootstrap values are shown on the trees.

Phylogenetic analyses were conducted for FMDV serotype A (Figure 5), serotype SAT 2 (Figure 6), and serotype O (Figure 7) using all available Ghanaian field isolates. For serotype A, the single sample (Gha_WEW-12) was compared with established topotype representatives, including isolates from Nigeria (2017, 2022) and Cameroon (2019). This isolate clustered within the Africa topotype, specifically the G-IV lineage, and showed closest genetic relatedness to two Nigerian 2017 and one Cameroonian 2019 G-IV isolates (Figure 5). For serotype SAT2 (Figure 6), the three isolates (Gha_G28, G32, and WEB1-17) clustered within SAT2 topotype V. They formed a distinct clade, separate from other topotype V isolates previously detected in Ghana (1990–1991), Togo (1990), Nigeria (1991), and West Africa (1990; AF426082). The four unique serotype O sequences were similarly evaluated against established topotype representatives. Gha_25 is grouped with the West Africa (WA) topotype and closely related to Lam/GHA/2012. (Figure 7). For the rest of the nine Ghana O isolates: Gha_LM x5 (representing five samples), Gha_x3 and Gha_20 are grouped with the East Africa 3 (EA-3) topotype and closely clustered with Cameroon 2019 and Nigeria 2020-2021 EA-3 isolates. Notably, Gha_x3 and Gha_20 formed a distinct clade within the EA-3 group, separate from other EA-3 isolates. (Figure 7).

### 3.2. Survey

**Figure 8:**
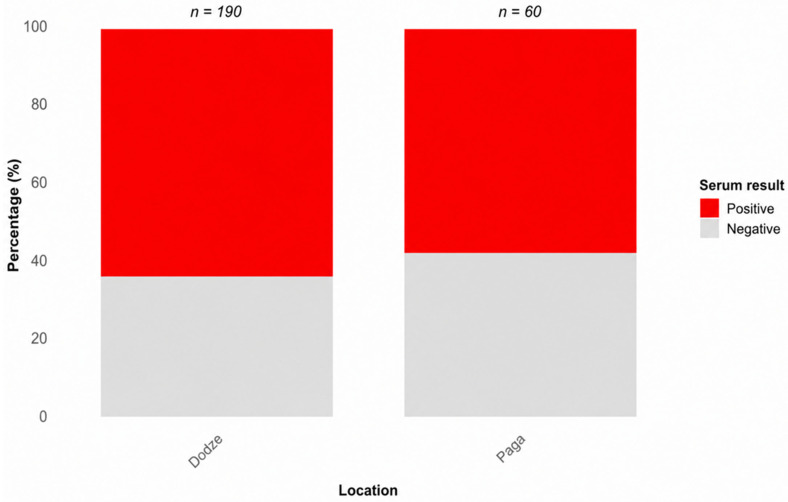
Distribution of sera collected from cattle from two Ghanaian entry points (Dodze and Paga) showing the number of foot-and-mouth disease (FMD) positive and negative samples. The bar chart illustrates the FMD-negative (grey), FMD-positive (red), samples collected from Dodze and Paga districts.

Overall, 156 of 250 (62.4%) samples from the points of entry tested positive for NSP antibodies. There was no significant difference in the positivity in Dzodze (121/190, 63.7%) and Paga (36/60. 60.0%), *p* = 0.55 (Figure 8). Only 6.7% of oropharyngeal samples using probang from Paga tested positive for FMDV. All probang samples from Dzodze tested negative for FMDV.

A higher proportion of positive samples was observed in both locations, Dzodze (south) and Paga (north), with Dodze showing a relatively larger sample size overall. These results highlight the variations in FMD seroprevalence and sampling intensity at the two entry points.

## 4. Discussion

This study identified FMDV serotypes causing disease outbreaks in Ghana in late 2024 and documented the carrier state of animals moving through Ghana’s borders, confirming the transboundary nature of the disease in western Africa. This situation could be worse due to livestock mobility through unapproved routes via nomadic activities, hampering the ability of Ghanaian veterinary authorities to conduct surveillance and implement control policies. This study aligns with The West African Roadmap to control and eliminate FMD, launched in 2016 [16]. In this initiative, 16 countries, including Ghana, collaborated to enhance epidemiological surveillance, diagnostic capacities, and vaccination strategies, aiming for regional disease elimination by 2027 [16].

One of the challenges in eradicating FMD in West Africa is the pastoral system of livestock management, whereby various animal species, including cattle, sheep, goats, and pigs, congregate in free-range feeding systems [17]. The Central and North Tongu districts of southern Ghana have significant livestock populations [18]. In these regions, livestock movement occurs regularly, with vendors engaging in farm-to-farm travel to purchase cattle or organize exchanges aligned with farmers’ requirements. Similar to other regions of Ghana, livestock are managed under extensive production systems. These practices create conditions for the introduction and spread of FMD in Central and North Tongu. FMD outbreaks historically occurred during the last quarter of the year [10]; however, recent observations have shown that cattle are now affected almost year-round.

The clinical manifestations identified in our study were predominantly localized around the oral and pedal regions of cattle, aligning with previous findings [19]. In contrast to high-yielding cattle, where FMD frequently results in a significant reduction in milk production due to udder lesions and systemic malaise [20], farmers in our study frequently reported low weight gain, grazing difficulties in affected animals, and mortality in young cattle, which could be due to myocarditis in newborn calves causing early congestive heart failure. These outcomes directly diminish household income and herd productivity. Furthermore, lesions on the teats and udder can induce pain during suckling, impeding calves from feeding effectively and predisposing them to malnutrition or mortality. Regrettably, many rural farmers seem to neglect udder lesions and their consequences, concentrating instead on the more conspicuous signs around the mouth and feet of the animals. The loss of a calf following nine months of gestation represents not only a biological setback but a substantial economic and emotional burden for rural households that rely almost exclusively on cattle rearing for their livelihood [21].

Phylogenetic analysis confirmed the presence of serotypes O, A, and SAT2 in Ghana, consistent with previous reports of multiple serotypes circulating both from outbreaks within four districts in southern Ghana [10] and across West Africa [22]. Within serotype O, one isolate (Gha_25) clustered closely with the West Africa (WA) topotype, whereas the remaining isolates grouped within the East Africa 3 (EA 3) topotype. Interestingly, Gha_x3 and Gha_20 formed a distinct clade within the EA 3 group, suggesting the emergence of a divergent lineage or potentially a new topotype. This pattern reflects the transboundary movement of FMDV, likely driven by livestock trade and cross border animal movement. These findings highlight the substantial genetic diversity of FMDV in endemic regions such as West Africa, where viral evolution is shaped by high rates of animal movement, porous borders, and limited control measures. They underscore the importance of continuous molecular surveillance and regular vaccine matching to support effective disease control. Comparative analyses of neighboring countries support this interpretation. The SAT 2 serotype has been identified in Nigeria [23,24] and Niger [3], showing significant divergence from the regional vaccine strains. Another significant finding is the detection of SAT2/V in Ghana where the viruses are most closely related to those from North Africa (Algeria 2023) and seems to give some evidence to support a West Africa origin of the viruses detected in Algeria. The presence of diverse topotypes in these countries indicates that cross-border livestock movement shapes the molecular epidemiology of FMDV in Ghana and other countries. This finding is significant for Ghana, as most livestock consumed in the country comes from neighboring countries such as Burkina Faso, Niger, and Nigeria. These animals are supplied to local abattoirs and are introduced into herds for rearing, creating opportunities for viral introduction and persistence. The lack of standardized screening at points of entry to the country implies that the serotype and topotype status of imported livestock may be unknown, posing risks for the transmission and local adaptation of new viral variants.

In Ghana, cattle are not routinely vaccinated against FMD, which is crucial for understanding the presence of the multiple FMDV serotypes and two different topotypes of FMDV serotype O. This suggests that viral evolution in the country primarily occurs in unvaccinated populations. The absence of vaccination pressure may allow for the persistence and undetected spread of diverse serotypes and topotypes, especially when compounded by the continuous introduction of livestock from neighboring countries. This highlights the dual challenges faced by Ghana and its subregions. While vaccine access and matching remain central issues, the lack of routine vaccination in Ghana increases the risk of uncontrolled transmission and underscores the urgency of implementing coordinated regional control measures.

## 5. Conclusions

The outbreak investigation in this study revealed the co-circulation of multiple FMDV serotypes implicated in the outbreak foci in Ghana. Apparently healthy cattle testing positive for FMD suggests the role of livestock mobility in the introduction and spread of the FMD virus in the country and beyond, and this highlights the need for enhanced molecular surveillance and coordinated cross-border control measures. Further studies are required to provide a better understanding of the transmission dynamics for surveillance and targeted control.

## Data Availability

All relevant data are in the paper.
